# Assessment of Ciguatera and Other Phycotoxin-Related Risks in Anaho Bay (Nuku Hiva Island, French Polynesia): Molecular, Toxicological, and Chemical Analyses of Passive Samplers

**DOI:** 10.3390/toxins12050321

**Published:** 2020-05-13

**Authors:** Mélanie Roué, Kirsty F. Smith, Manoella Sibat, Jérôme Viallon, Kévin Henry, André Ung, Laura Biessy, Philipp Hess, Hélène Taiana Darius, Mireille Chinain

**Affiliations:** 1 Institut de Recherche pour le Développement, UMR 241 EIO, 98702 Faa’a, Tahiti, French Polynesia; 2 Institut Louis Malardé, UMR 241 EIO, 98713 Papeete, Tahiti, French Polynesia; jviallon@ilm.pf (J.V.); khenry@ilm.pf (K.H.); aung@ilm.pf (A.U.); tdarius@ilm.pf (H.T.D.); mchinain@ilm.pf (M.C.); 3 Cawthron Institute, Nelson 7042, New Zealand; kirsty.smith@cawthron.org.nz (K.F.S.); laura.biessy@cawthron.org.nz (L.B.); 4 Ifremer, DYNECO, 44000 Nantes, France; manoella.sibat@ifremer.fr (M.S.); philipp.hess@ifremer.fr (P.H.)

**Keywords:** ciguatera monitoring, *Gambierdiscus*, ciguatoxins, SPATT passive samplers, HP20 resin, CBA-N2a, LC-MS/MS, WS artificial substrate, qPCR, HTS metabarcoding

## Abstract

Ciguatera poisoning is a foodborne illness caused by the consumption of seafood contaminated with ciguatoxins (CTXs) produced by dinoflagellates from the genera *Gambierdiscus* and *Fukuyoa*. The suitability of Solid Phase Adsorption Toxin Tracking (SPATT) technology for the monitoring of dissolved CTXs in the marine environment has recently been demonstrated. To refine the use of this passive monitoring tool in ciguateric areas, the effects of deployment time and sampler format on the adsorption of CTXs by HP20 resin were assessed in Anaho Bay (Nuku Hiva Island, French Polynesia), a well-known ciguatera hotspot. Toxicity data assessed by means of the mouse neuroblastoma cell-based assay (CBA-N2a) showed that a 24 h deployment of 2.5 g of resin allowed concentrating quantifiable amounts of CTXs on SPATT samplers. The CTX levels varied with increasing deployment time, resin load, and surface area. In addition to CTXs, okadaic acid (OA) and dinophysistoxin-1 (DTX1) were also detected in SPATT extracts using liquid chromatography coupled to tandem mass spectrometry (LC-MS/MS), consistent with the presence of *Gambierdiscus* and *Prorocentrum* species in the environment, as assessed by quantitative polymerase chain reaction (qPCR) and high-throughput sequencing (HTS) metabarcoding analyses conducted on passive window screen (WS) artificial substrate samples. Although these preliminary findings await further confirmation in follow-up studies, they highlight the usefulness of SPATT samplers in the routine surveillance of CP risk on a temporal scale, and the monitoring of other phycotoxin-related risks in ciguatera-prone areas.

## 1. Introduction

Harmful algal blooms (HABs) cause major sanitary, environmental, and economic problems in many aquatic environments worldwide [1]. Numerous countries thus maintain routine monitoring programs for the early detection of HAB events and the presence of associated toxins in order to mitigate the associated impacts. Most of these programs are based on the direct monitoring of potentially toxic microalgal and cyanobacterial species and qualitative/quantitative analyses of toxins accumulated in marine products intended for human consumption [2,3]. Since 2004, the Solid Phase Adsorption Toxin Tracking (SPATT) technique, which enables the passive sampling of dissolved toxins present in the water column associated with episodic HAB events, has increased in popularity as a complementary method [4]. Numerous laboratory- and field-based studies have demonstrated the ability of this technique to detect a large array of lipophilic and hydrophilic toxins produced by various microalgae and cyanobacteria, in both marine and freshwater environments [5,6]. SPATT samplers contain porous synthetic resins, and of the many adsorbent substrates tested to date, the DIAION^®^ HP20 resin, an aromatic type adsorbent based on a cross-linked polystyrene matrix, appears to be the most versatile, and thus most used, substrate [6].

Ciguatera poisoning (CP) is a seafood-borne illness caused by the consumption of tropical marine seafood products (i.e., fish and invertebrate species) contaminated with ciguatoxins (CTXs) [7]. These polyether neurotoxins are produced by benthic dinoflagellates belonging to the genera *Gambierdiscus* and *Fukuyoa* [8]. Ciguatera is endemic to coral reef areas in the Caribbean Sea and Pacific and Indian Oceans [9]; however, its expansion into previously unaffected temperate regions has been documented since 2004 [10,11]. This rise of CP prevalence worldwide has prompted increased research efforts, most notably towards the improvement of current monitoring methods to predict and manage outbreaks. The suitability of SPATT samplers for the in situ monitoring of CTXs was recently assessed in a ciguatera hotspot in French Polynesia (Anaho Bay, Nuku Hiva Island, Marquesas archipelago) [12]. Previous studies in Anaho Bay have shown the recurrent presence of *Gambierdiscus* communities with the co-occurrence of at least five species and the predominance of *G. polynesiensis* [13,14,15], a species known to be a high producer of Pacific CTXs (P-CTXs) [16,17]. Moreover, P-CTXs were detected in fish [13], as well as in trochus [14] and sea urchins [15] collected from Anaho Bay. Finally, both trochus and sea urchin specimens were recently involved in atypical CP cases [15,18]. P-CTXs were successfully detected in methanol extracts of SPATT devices containing 10 g of HP20 resin, following a two-day deployment in Anaho Bay in July 2015 [12]. The presence of P-CTXs in these samplers was concomitant with the observation of *Gambierdiscus polynesiensis* populations [15], as well as the detection of P-CTXs in sea urchins [15]. These results confirmed the potential of SPATT technology as a passive environmental surveillance tool to support CP risk assessments and management programs, particularly in areas at high risk of ciguatera [12]. SPATT samplers—which are known for their relatively low cost as well as simplicity of preparation, deployment, transport, and storage [19]—appear well-suited for monitoring CTXs in remote and widely dispersed island locations, such as Nuku Hiva Island, and, more globally, other Pacific Islands Countries and Territories (PICTs), whose populations are the most vulnerable to CP [20].

The present work is a follow-up study to previous investigations conducted by Roué, et al. [12] in the Anaho Bay. To this end, two successive field experiments were conducted in the same study site using SPATT technology in an attempt to (i) follow the dissolved CTX trend in the environment over a 22 month period, and (ii) study the effects of different deployment times and device formats (i.e., resin load and surface of exposure) on the overall adsorption efficacy of SPATT samplers towards CTXs. Practically, SPATT devices of various surface areas, filled with 2.5 or 5 g of HP20 resin, were deployed in Anaho Bay in November 2016 and August 2018 for one, three, or six days. The levels of CTXs in methanol extracts were assessed using the mouse neuroblastoma cell-based assay (CBA-N2a), and the identity of congeners was determined using liquid chromatography coupled to tandem mass spectrometry (LC-MS/MS). In parallel, the abundance of specific *Gambierdiscus* species in Anaho Bay was assessed through quantitative polymerase chain reaction (qPCR) assays conducted on passive window screen (WS) artificial substrate samples. Finally, in order to further characterize the potential human health risks associated with the consumption of marine products from Anaho Bay, the composition of benthic dinoflagellate assemblages and presence of related toxic compounds in this area were also assessed using the (i) high-throughput sequencing (HTS) metabarcoding of WS samples and (ii) LC-MS/MS based multi-toxin screening of SPATT samples, respectively.

## 2. Results

### 2.1. Molecular Analyses of Environmental Samples

Benthic/epiphytic dinoflagellate cells were collected from macroalgal substrates as well as WS artificial substrates during November 2016 and August 2018 field campaigns in Anaho Bay.

Microscopic observations revealed the presence of *Gambierdiscus*, *Ostreopsis*, and *Prorocentrum* cells on macroalgal substrates collected in November 2016, whereas only *Gambierdiscus* and *Ostreopsis* cells were observed in August 2018 (Table 1).

Semi-quantitative, species-specific qPCR assays were performed on WS samples to determine the *Gambierdiscus* spp. relative abundance and species distribution. A reduction in *Gambierdiscus* cell abundance was observed between November 2016 and August 2018, with *Gambierdiscus* cells no longer detected in the 2018 samples (Table 1). Among the five species detected in November 2016, *G. polynesiensis* was the dominant species, followed by *G. carpenteri* (Table 1).

**Figure 1:**
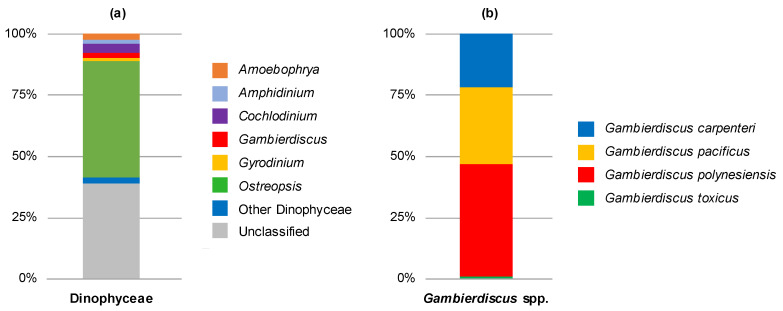
Relative abundances of (**a**) Dinophyceae reads at the genus level and (**b**) *Gambierdiscus* reads at the species level from window screen samples collected in Anaho Bay in August 2018 (n = 5). All the classified genera representing less than 1% of all Dinophyceae reads (Table S1) were identified as “other Dinophyceae”.

In order to fully characterize the composition of benthic assemblages in *Gambierdiscus* and other HAB species, HTS metabarcoding analyses were carried out on artificial WS samples from August 2018. Sequencing targeting the large subunit ribosomal RNA (LSU) D1-D2 region generated between 27,069 and 97,610 Dinophyceae reads per sample after quality filtering, size trimming, and chimera detection (Table S1). Only 2% of the reads corresponded to *Gambierdiscus* species (Figure 1a), with four species identified: *G. polynesiensis* was the dominant species, representing 46% of *Gambierdiscus* reads, followed by *G. pacificus* (31%), *G. carpenteri* (22%), and *G. toxicus* (1%) (Figure 1b, Table S1). The most abundant Dinophyceae genus detected was *Ostreopsis*, representing nearly 48% of all Dinophyceae reads (Figure 1a), with three species identified: *O.* cf. *ovata*, *O.* cf. *lenticularis*, and *O.* cf. *rhodesiae* (Table S1).

In addition, other known toxin-producing genera were detected, e.g., *Amphidinium*, *Azadinium*, *Coolia*, *Dinophysis*, *Karenia*, and *Prorocentrum*, although the numbers of reads for these taxa were low (Table S1). Bayesian phylogenetic analyses showed that all taxa classifications using the custom dinoflagellate sequence databases were correct, although the short sequences (approximately 350 bp) were not able to resolve some deeper phylogenetic relationships (Figure S1). Some taxa were able to be classified to the species level, in particular, *Gambierdiscus* spp., while other groups could not be successfully resolved. For example, some *Ostreopsis* spp. and *Amphidinium* spp. were not able to be assigned to specific species (Figure S1) either because they represent undescribed species or species that are not present in the GenBank database. Therefore, some taxa have been assigned to the genus-level only, or “cf.” has been used to indicate some uncertainty with the classification.

### 2.2. Evaluation of SPATT Samples Toxicity Using CBA-N2a

**Figure 2:**
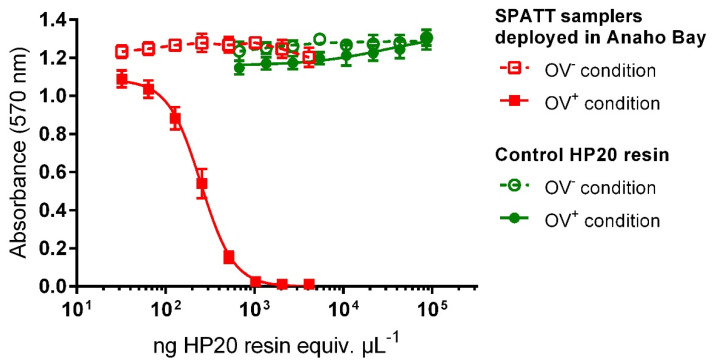
Dose–response curves for neuroblastoma (N2a) cells exposed, in the absence (OV^−^ condition) and presence (OV^+^ condition) of ouabain and veratridine, to increasing concentrations of methanol extracts (expressed in pg dry extract µL^−1^ and then converted to ng HP20 resin equiv. µL^−1^) from control HP20 resin or Solid Phase Adsorption Toxin Tracking (SPATT) devices (n = 3) filled with 2.5 g of HP20 resin and deployed for three days in Anaho Bay in November 2016. Data represent the means ± standard errors (SE) of values obtained from three independent neuroblastoma cell-based assays (CBA-N2a), each point run in triplicate.

Regardless of the resin load (2.5 or 5 g of HP20 resin), deployment time (1, 3, or 6 days), or surface area (44 or 71 cm^2^) of the SPATT devices deployed in Anaho Bay in November 2016 and August 2018, all methanol extracts were found to be toxic toward neuroblastoma (N2a) cells, with activity typical for CTXs: cytotoxic effects were observed in the presence of ouabain (O) and veratridine (V) (i.e., OV^+^ conditions), whereas no cytotoxicity was detected in the absence of O and V (i.e., OV^−^ conditions) (Figure 2).

Mean half-maximal effective concentration (EC_50_) values ranging from 614 ± 123 to 6319 ± 1237 pg of dry extract µL^−1^ were obtained. These raw EC_50_ values were then converted into quantities of resin (ng HP20 resin equiv. µL^−1^) to be further compared with each other whatever the amount of resin used (i.e., 2.5 or 5 g), which ranged from 270 ± 51 to 1.5 × 10^4^ ± 0.5 × 10^4^ ng HP20 resin µL^−1^ (Table 2). Corresponding toxin contents were estimated at between 0.14 ± 0.04 and 7.0 ± 1.2 ng P-CTX3C equiv. g^−1^ HP20 resin (Table 2).

**Figure 3:**
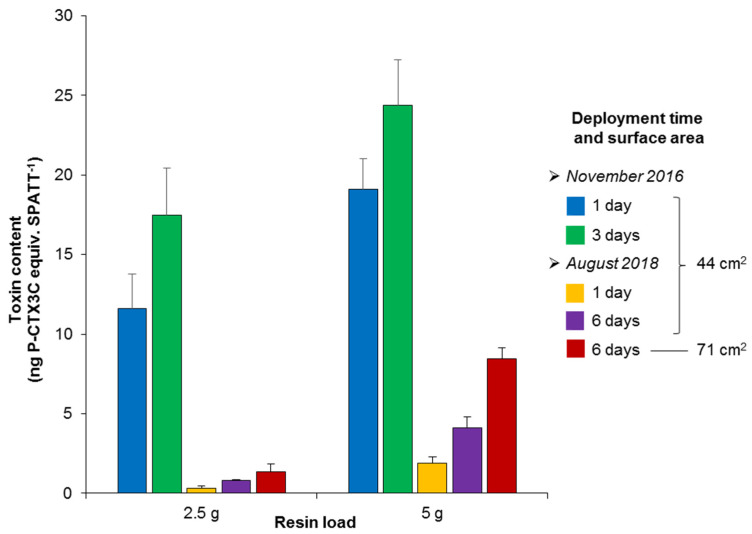
Toxin contents adsorbed on SPATT devices deployed in Anaho Bay in November 2016 and August 2018, based on the CBA-N2a data detailed in Table 1. Error bars represent SE.

When the toxin contents were calculated according to the total quantity of resin per SPATT device, i.e., 2.5 or 5 g, these toxin contents varied from 0.34 ± 0.10 to 23.5 ± 3.2 ng P-CTX3C equiv. SPATT^−1^ (Figure 3). The toxicity data indicated a significant decrease (*p* < 0.0001) in CTX levels in Anaho Bay in 2018 compared to 2016; indeed, the amounts of CTXs detected on SPATT devices from August 2018 were 10- to 34-fold lower than those from November 2016, for a deployment time of 24 h and resin loads of 5 and 2.5 g, respectively (Figure 3).

Concerning the effect of resin load, whatever the period of study, time of deployment, and surface of exposure, the use of 5 g of HP20 resin led to the adsorption of 1.4- to 6.2-fold higher levels of CTXs compared with a resin load of 2.5 g (Figure 3).

As for the deployment time, 44 cm^2^ SPATT devices deployed for three and six days accumulated 1.3- to 1.5- and 2.1- to 2.3-fold more CTXs than the ones deployed for one day, respectively, with the highest increases with deployment duration being observed for resin loads of 2.5 g (Figure 3).

Finally, regarding the sampler surface (only tested in 2018), after six days of deployment, the amounts of CTXs contained in larger SPATT devices were found to be 1.7- and 2.1-fold higher than in smaller SPATT samplers for resin loads of 2.5 and 5 g, respectively (Figure 3).

### 2.3. Characterization of Toxins Adsorbed by HP20 Resin Using LC-MS/MS

**Figure 4:**
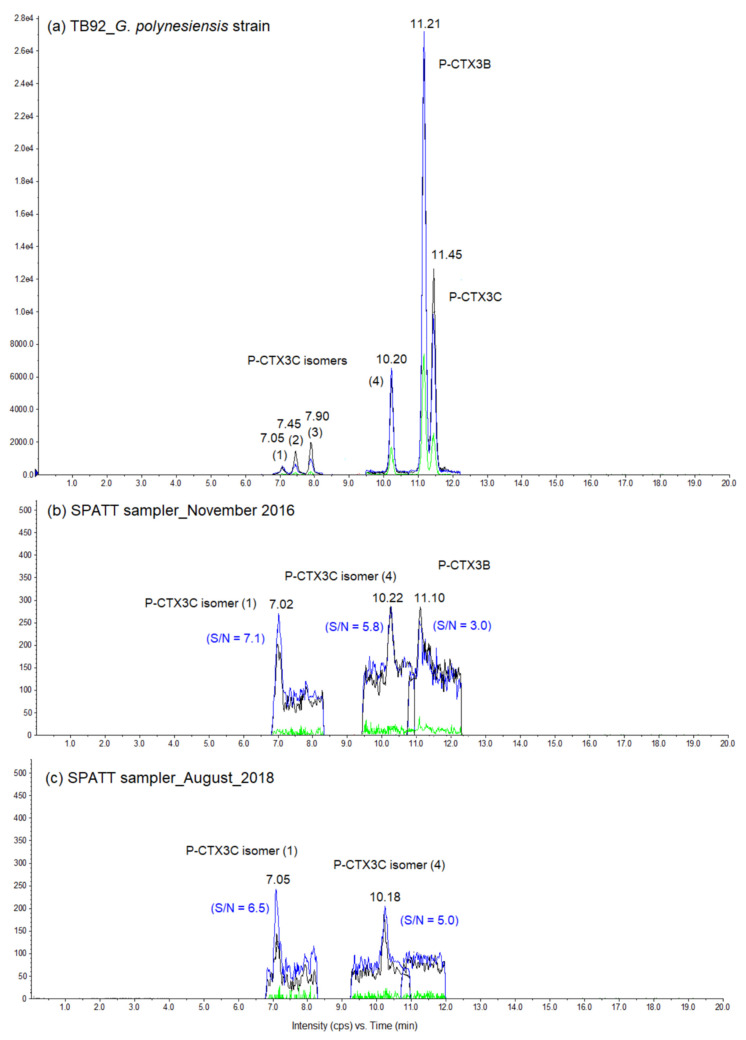
Liquid chromatography coupled to tandem mass spectrometry (LC-MS/MS) chromatograms of methanol extracts from (**a**) TB92-*Gambierdiscus polynesiensis* strain, and (**b**,**c**) SPATT devices filled with 5 g of HP20 resin and deployed for 24 h in Anaho Bay in November 2016 and August 2018, respectively. Chromatograms were acquired in positive scheduled multi-reaction mode (MRM) mode with a retention window of 90 sec, representing the MRM transitions of P-CTX3B/C at *m/z* 1023.5 > 1005.6 (in blue), 1040.6 > 1005.6 (in black), and 1023.6 > 125.1 (in green). The signal-to-noise (S/N) was calculated with three standard deviations for the *m/z* transition 1023.6 > 1005.6. The two potential P-CTX3C isomers were tentatively identified on the basis of two MRM transitions and the correct retention time of these isomers compared to that of the highly toxic *G. polynesiensis* strain TB92.

All methanol extracts from SPATT devices deployed in Anaho Bay in November 2016 and August 2018 were analyzed using a LC-MS/MS based multi-toxin screening approach, leading to the detection of two groups of marine toxins. The presence of P-CTXs was confirmed in all samples, although at non-quantifiable concentrations (limit of quantification of P-CTX3C: 6 ng/mL). Two uncharacterized P-CTX3C isomers, consistently present in methanol extracts of TB92—a highly toxic strain of *Gambierdiscus polynesiensis* (Figure 4a)—were also detected in SPATT samples collected in November 2016 and August 2018 (Figure 4b,c), whereas P-CTX3B was found only in November 2016 samples (Figure 4b).

**Figure 5:**
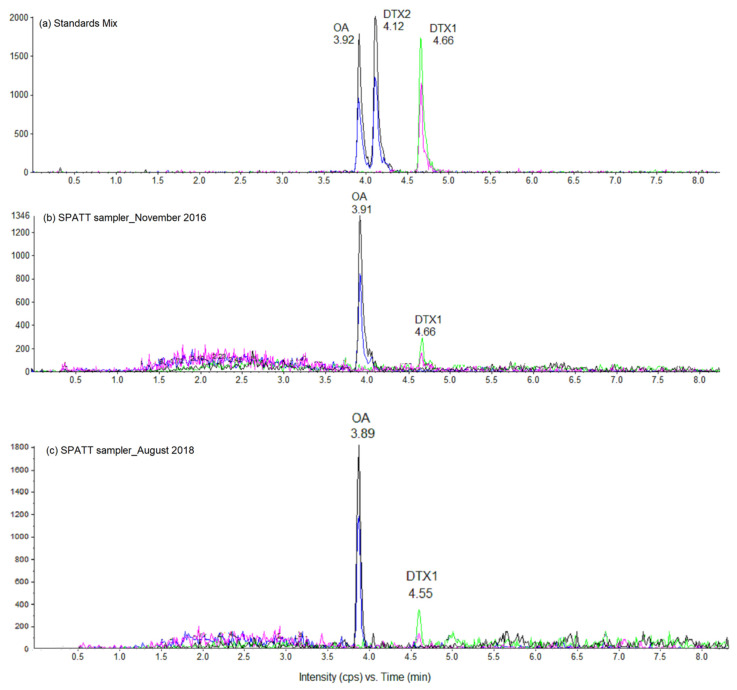
LC-MS/MS chromatograms of (**a**) okadaic acid (OA), dinophysistoxin-1 (DTX1), and dinophysistoxin-2 (DTX2) standards mix solution (NRC certified reference material); and (**b**,**c**) methanol extracts from SPATT devices filled with 5 g of HP20 resin and deployed in Anaho Bay for three days in November 2016 and for six days in August 2018, respectively. Chromatograms were acquired in negative MRM mode on *m/z* transitions 803.4 > 255.1 (black) and 803.4 > 113.5 (blue) for OA and its analog DTX2, as well as on *m/z* transitions 817.5 > 257.9 (green) and 817.5 > 112.9 (pink) for DTX1.

Among the other toxins sought, okadaic acid (OA) and dinophysistoxin-1 (DTX1) were also detected in all SPATT devices for OA, depending on the deployment time for DTX1 (Figure 5).

The toxin contents of OA per SPATT device ranged from 3.3 to 47.0 ng OA SPATT^−1^ (Table 3). For similar conditions, an increase in the time of exposure, resin load, or surface area led to 1.4- to 5.6-, 1.4- to 2.9-, or 1.4- to 1.9-fold higher levels of OA, respectively (Table 3). For a 24 h deployment time, a 3-fold decrease in OA contents was observed between November 2016 and August 2018.

Low toxin contents of DTX1 per SPATT device were detected, ranging from 2.0 to 6.9 ng DTX1 SPATT^−1^, provided that SPATT devices were deployed for at least three days (Table 3). As observed for OA, the highest level of DTX1 was detected after six days of exposure in August 2018 using 71 cm^2^ SPATT devices filled with 5 g of HP20 resin.

None of the other marine biotoxins sought (see Section 5.6 for details) were detected through LC-MS/MS multi-toxin screening analyses.

## 3. Discussion

### 3.1. Benthic Harmful Algae Populations and Toxin Profiles over Time in Anaho Bay

This study confirmed the ciguateric status of Anaho Bay. Indeed, the CBA-N2a toxicity data showed that dissolved CTXs previously detected on SPATT samplers deployed in the area in July 2015 [12] were still present in the water column at decreasing, yet quantifiable, amounts in November 2016 and August 2018. Molecular analyses of WS samples showed that *G. polynesiensis*, a species known to be highly toxic [16,17], was the dominant *Gambierdiscus* species both in November 2016 [14] and August 2018, although the abundance of total *Gambierdiscus* cells was low, especially in August 2018. The concentrations of CTXs detected on SPATT samplers after 24 h of deployment in August 2018 were significantly lower than in November 2016, consistent with the qPCR data for WS samples indicating a reduction in *Gambierdiscus* cells between November 2016 and August 2018. In addition, HTS metabarcoding analyses of environmental samples confirmed the very low abundance of *Gambierdiscus* in August 2018, in comparison with other dinoflagellate genera, in particular, *Ostreopsis*. Similarly, a two- to three-fold decrease in the levels of CTXs detected in locally sourced marine invertebrates was observed between November 2016 [14,15] and August 2018 (i.e., 0.15 and 0.19 ng P-CTX3C equiv. g^−1^ of tissue for sea urchins and trochus, respectively; unpublished data). Finally, the LC-MS/MS data showed that P-CTX3B, as well as two uncharacterized P-CTX3C isomers (tentative identification), were detected on SPATT devices deployed in November 2016, whereas only the two potential P-CTX3C analogs were present in the August 2018 samples. The detection of fewer CTX analogs in the SPATT devices in August 2018 vs. November 2016 could be due to the lower levels of total P-CTXs, as confirmed by the CBA-N2a results. As observed for trochus or sea urchin, the number of analogs detected decreased when the amount of total P-CTXs decreased [14,15]. Another explanation could be that uncharacterized P-CTX3C analogs are more stable in the water column and thus take longer to degrade than P-CTX3B; however, this hypothesis still needs to be confirmed.

The LC-MS/MS multi-toxin screening approach resulted in the detection of dissolved OA and DTX1 in Anaho Bay for the first time (in both November 2016 and August 2018). This result is consistent with the concomitant detection of *Prorocentrum* cells, by both microscopic observation and HTS metabarcoding analyses of environmental samples, although at very low abundance at the time of the field campaigns. Dinoflagellates of the genus *Prorocentrum* are potential producers of diarrheic shellfish poisoning (DSP) toxins [21]. Traces of *Dinophysis*, another potential producer of DSP toxins [21], were also detected in Anaho Bay in August 2018 using HTS metabarcoding. The low levels of OA and DTX1 (ng order) detected on the SPATT devices are consistent with the low number of reads detected for *Prorocentrum* (0.35%) and the very low number of reads detected for *Dinophysis* (0.03%). In bloom scenarios, much higher concentrations of OA-group toxins are typically detected in such SPATT devices, e.g., as was the case in France in 2016 [22]. The low concentrations of OA-group toxins are coherent with the low numbers of *Prorocentrum* cells in Anaho Bay, and it is not clear whether these were in decline, similarly to *Gambierdiscus* cells, or had been low over longer periods. In any case, the presence of OA and DTX1 in Anaho Bay may have contributed to the gastrointestinal symptoms reported by patients following the consumption of marine invertebrates from Anaho Bay [15,18], even though detectable amounts of these two toxins could not be positively confirmed in trochus and sea urchins analyzed one month after the poisoning occurred [14,15].

Conversely, neither palytoxin nor its analogs (i.e., ovatoxins) were found in SPATT samples by LC-MS/MS despite the high predominance of *Ostreospsis* spp. in Anaho Bay. This result is consistent with recent findings that both *O. lenticularis* [23] and *O.* cf. *ovata* (pers. comm., M. Chinain and D. Réveillon) strains from French Polynesia show no toxicity (as assessed by CBA-N2a and LC-MS/MS analyses), whereas strains of both species were found to be toxic in the Caribbean and Mediterranean regions [21,24]. However, the reporting of other potentially toxic HAB genera, such as *Amphidinium*, *Azadinium*, *Coolia*, *Karenia*, and *Ostreopsis* [21] in Anaho Bay emphasizes the importance of extending the environmental surveillance in this CP-endemic area to toxin groups other than CTXs.

Using the LC-MS/MS multi-toxin screening approach, several other biotoxin groups were screened for but were not detected, because of either the absence or low levels of the producing dinoflagellates in Anaho Bay. As an example, the non-detection of cyclic imines and azaspiracids is consistent with the very low number or absence of metabarcoding reads for the genera *Alexandrium*, *Azadinium*, *Karenia*, and *Vulcanodinium* (0% to 0.01% of the total Dinophyceae reads).

### 3.2. Effects of Deployment Time on the Efficacy of CTX Detection

To date, there is no consensus about the minimum/optimal deployment time of SPATT devices in the field and, as highlighted by Kudela [5], the deployment duration usually depends on the research or monitoring design of the programs. The present study provides some preliminary data about the potential of SPATT samplers to adsorb different amounts of CTXs under varying deployment times. In most of the field studies reported in the literature, SPATT samplers were deployed for one week [25,26,27,28,29,30], but sometimes for longer periods (up to one month) [31,32]. However, from a practical point of view, a deployment time of several days/weeks may be difficult or even impossible to implement, most notably in remote areas that are difficult to access, as is the case for Anaho Bay or many ciguatera-prone areas in PICTs. In the present study, using a highly sensitive detection method such as the CBA-N2a, the results showed that CTXs could be detected on SPATT samplers deployed in a long-standing ciguatera hotspot after only 24 h of deployment time. To the best of our knowledge, only one laboratory study has investigated the effect of different sorbent materials on SPATT adsorption efficiency [33], and two field studies have previously documented the successful monitoring of various toxin groups using limited deployment times; detectable amounts of OA, DTX1, yessotoxin (YTX), and pectenotoxins (PTXs) were found on HP20 SPATT devices deployed for only 3.5 h at Wedge point, New Zealand [4], while anatoxin-a and homoanatoxin-a were successfully detected on Strata-X^TM^ SPATT devices exposed for only 4 h in Waipoua River, New Zealand [34].

Most importantly, using longer deployment times—i.e., three and six days—led to the adsorption of higher levels of CTXs onto HP20 resin. However, the highest level of CTXs adsorbed per gram of HP20 resin (i.e., 7 ng P-CTX3C equiv. g^−1^ HP20 resin) was nearly 8-fold lower than the saturation rate previously determined under laboratory conditions by Roué, et al. [12] (i.e., 55 ng P-CTX3C equiv. g^−1^ HP20 resin for a deployment of 10 g of HP20 resin during 48 h in an in vitro culture of TB92, a highly toxic *G. polynesiensis* strain). These findings suggest that SPATT devices deployed in Anaho Bay could potentially have retained higher amounts of CTXs if they had been deployed for a longer period, provided the presence of higher concentrations of CTXs in the environment. Likewise, the results also showed an increase in OA adsorption with increasing deployment times but with maximum levels per gram of HP20 resin lower than those reported in other studies [22,29], suggesting the limit of saturation of the SPATT has not been reached in the field experiments.

The results from the present study showed that a time of exposure of at least three days was necessary to detect DTX1. A previous study had already demonstrated a linear increase in the quantities of several polyether biotoxins adsorbed by HP20 resin for at least the first eight days of exposure at Wedge point, New Zealand [4]. Taken together, all these observations are consistent with laboratory studies suggesting that HP20 resin is a slow accumulator of various toxins as compared with other adsorbent substrates (e.g., Sepabeads^®^, Oasis^®^ HLB, or Strata-X resins) and is thus more appropriate for long exposure periods [33,35,36]. Consequently, when low concentrations of toxins are expected to circulate in the water column (i.e., in areas with low-to-moderate CP risk), using longer deployment times (i.e., ≥1 week) could prove useful for increasing the detectability of toxins. Similarly to other kinetic samplers, if the uptake kinetic of SPATT devices is faster than their offload kinetic, toxin adsorption would increase linearly under prolonged exposure [5]. However, this is not a general rule for all toxins; for example, a fast desorption rate of paralytic shellfish poisoning (PSP) toxins from SP700 resin was observed in toxin-free seawater, indicating that for these hydrophilic toxins, the field deployment of SPATT samplers should not exceed seven days to avoid toxin loss [37]. Furthermore, it should be noted that a longer deployment time is likely to increase biofouling on SPATT devices, thus reducing water flow through the resin and toxin adsorption, although biofouling may play less of a role in tropical oligotrophic areas than in temperate eutrophic areas.

### 3.3. Effects of Resin Load on the Efficacy of CTX Detection

No consensus currently exists on the minimal/optimal resin load to use in SPATT samplers. In the literature, the resin loads tested in the different studies ranged from 0.3 g [22,33] to 10 g [12,27], although the amount most commonly used is between 3 and 5 g [4,25,28,30,31,32,36]. To the best of our knowledge, only one field-study by Zendong, et al. [22], conducted in the Mediterranean Sea, has compared the efficacy of SPATT devices to detect several lipophilic phycotoxins when filled with different resin loads (i.e., 0.3, 3, and 10 g of HP20 resin). The authors concluded that 3 g of resin offered the best compromise in terms of cost (lesser resin and solvent consumption), adsorption capacity, and efficacy (lesser potential for clogging) [22]. They noted that the 69 cm^2^ exposure surface used in their study was insufficient to avoid the superimposition of resin particles for a 10 g resin load, leading to a decrease in toxin adsorption efficiency. The results obtained in the present study are thus consistent with previous findings by Zendong et al. [22], as SPATT devices filled with 5 g of HP20 resin always led to the adsorption of higher levels of P-CTXs, OA, and DTX1 per device than with 2.5 g. The same observation applies when larger devices were used, i.e., 71 vs 44 cm^2^. These findings suggest that the contact surface between resin and seawater was relatively well optimized in the 44 cm^2^ SPATT samplers filled with 2.5 g and that 71 cm^2^ devices should be used for resin loads of 5 g in order to allow a better distribution of the resin (or higher surface/resin weight ratio) and thus a higher toxin adsorption.

## 4. Conclusions

This study provides preliminary data on the modular use of the SPATT technique (HP20 resin)—i.e., different deployment time, resin loads and surface areas—for the in situ monitoring of CTXs in CP-prone areas, as well as the survey of additional HAB-related toxin groups potentially present in ciguateric biotopes. In the Anaho ciguatera hotspot, the deployment of 44 cm^2^ SPATT devices filled with 2.5 g of HP20 resin for only 24 h allowed the assessment of a decrease in CTX concentrations over a 22 month time period. The results obtained over longer deployment times led us to speculate that in areas where the ciguatera risk is unknown or considered low-to-moderate, increasing the amount of resin and time of deployment could help improve toxin detection, provided the surface of the device is optimized to avoid resin clogging. From a practical point of view, it is suggested that when the concentrations of dissolved toxins are potentially low, the deployment of 71 cm^2^ SPATT samplers filled with 5 g of resin for at least one week could be considered.

More widely, both the design of the passive sampler (e.g., adsorbent substrate, resin load, and device surface) and deployment times should be adapted to the characteristics of the study site (e.g., the accessibility, hydrodynamics, trophic state, and diversity of HAB communities) and the targeted toxin(s) (e.g., lipophilic or hydrophilic toxins). Similarly to that of other environmental passive samplers, the calibration of SPATT samplers is problematic and is perhaps the single greatest issue in current efforts to achieve better acceptance of this technology in HAB monitoring and management programs [5]. While waiting for the inclusion of this useful tool in the context of routine monitoring programs, the use of SPATT technology in combination with classic monitoring techniques (e.g., microalgal cell enumeration/identification or the quantification of toxins in fish/shellfish) should be strongly encouraged, in order to gain better knowledge of the extent of toxin contamination of aquatic environments.

## 5. Materials and Methods

### 5.1. Environmental Analyses

The study site is located in Anaho Bay (Nuku Hiva Island, Marquesas archipelago, French Polynesia; 08°49.171′ S, 140°03.923′ W). Anaho Bay is a remote area, difficult to access from the main island of Tahiti (i.e., several hours’ travel by plane, 4 × 4 car, and boat) and field campaigns cannot be regularly conducted.

Environmental samples of phytobenthos were collected from Anaho Bay in November 2016 and August 2018, using both natural (i.e., macroalgae) and artificial (i.e., window screen, WS) substrate methods, as previously described by Darius et al. [14]. The presence of *Gambierdiscus*, *Ostreopsis*, and *Prorocentrum* cells was assessed microscopically in macroalgal samples.

Semi-quantitative, species-specific qPCR assays were performed on WS samples to assess the relative cell abundance and distribution of the following *Gambierdiscus* species: *G. polynesiensis*, *G. toxicus*, *G. pacificus*, *G. australes, G. caribaeus*, and *G. carpenteri*, which are currently reported in French Polynesia, as well as four additional species/phylotypes, i.e., *G. belizeanus*, *G. carolinianus*, *G. ruetzleri*, and *Gambierdiscus* ribotype 2. The qPCR assays were performed using the protocol previously described in Vandersea et al. [38] and Darius et al. [14], with a sensitivity of detection of the assays established at approximately 10 cells, i.e., ranging from ≈ 20 to 5000 gene copy numbers depending on the species targeted [38]. The same procedure was followed for the taxonomic identification at the species level of the eight clonal cultures of *Gambierdiscus* further established from field samples.

Additionally, the WS samples collected in August 2018 were further analyzed using HTS metabarcoding, using primers targeting the LSU D1-D2 region, as previously described by Smith et al. [39]. Briefly, samples were centrifuged, and DNA was extracted from the pellets using Qiagen DNeasy PowerSoil kits (Qiagen, Carlsbad, CA, USA). The primers (D1R-F and D3B-R) were modified to include Illumina^TM^ overhang adaptors. Libraries were prepared using the Illumina^TM^ two-step PCR amplicon library preparation method and sequenced using an Illumina^TM^ MiSeq sequencer with 2 × 250 bp paired-end reads at Auckland Genomics (University of Auckland, Auckland, New Zealand). All data generated were quality checked using the following tools: FastQC, FastQscreen, and SolexaQA [40]. Further analyses were carried out in Mothur v1.37.6 [41] as described by Smith et al. [42]. Phylogenetic analyses were carried out in Geneious^®^ using MrBayes 3.1.2 [43].

### 5.2. HP20 Resin and SPATT Device Preparation

Adsorbent styrene-divinylbenzene resin (Diaion^®^ HP20, Supelco, USA) was activated overnight by stirring at a low speed in methanol (MeOH) at a 10:1 ratio (mL g^−1^). The HP20 resin was then filtered under vacuum using a fritted funnel and, subsequently, thoroughly rinsed with de-ionized (MilliQ) water to remove any methanol residues. The hydrated resin was kept in MilliQ water at a 10:1 ratio (mL g^−1^) at 4 °C until the preparation of the SPATT devices.

SPATT samplers consisted of two layers of 100 µm nylon mesh filled with HP20 resin (2.5 or 5 g wet weight) and clipped between either two PVC circular disks (7.5 cm in diameter, corresponding to a surface of 44 cm^2^) or embroidery frames (9.5 cm diameter, corresponding to a surface of 71 cm^2^). SPATT devices were set up shortly before the field experiments (i.e., less than 1 week before) and were kept in MilliQ water at 4 °C until in situ deployment.

### 5.3. Field Deployments of SPATT Devices

On-site, SPATT devices were placed in plastic grids to prevent damage from fish grazing and then maintained in a vertical position in the water column using weights and floats. The deployment occurred near dead coral blocks colonized by macroalgae likely to shelter benthic *Gambierdiscus* cells, in locations with a moderate current to allow the circulation of seawater through the HP20 resin. Two series of experiments were conducted: one in November 2016, where 44 cm^2^ SPATT devices were filled with 2.5 and 5 g of HP20 resin and retrieved after one or three days of deployment (Table 1); and the second one in August 2018, where 44 and 71 cm² SPATT devices were filled with 2.5 and 5 g of HP20 resin and retrieved after one or six days of deployment (Table 1). Each condition was tested in triplicate (n = 30 SPATT devices). A total of 6 samplers could not be retrieved due to damage or loss. Following collection, SPATT devices were stored at 4 °C in natural seawater until toxin extraction.

### 5.4. Toxin Extraction from HP20 Resin

Each SPATT device was rinsed thoroughly with MilliQ water, in order to remove any cell debris or epiphytes possibly adhering to the nylon mesh, and then carefully dismantled to retrieve the HP20 resin. Using a fritted funnel under vacuum, the resin was washed thoroughly with MilliQ water in order to eliminate salt residues. Toxins were extracted from HP20 resin with MeOH in a 10:1 ratio (mL·g^−1^). The resulting methanol extracts were dried under vacuum at 40 °C using a rotary evaporator (Rotavapor RII, Büchi, Switzerland) and stored at 4 °C until CBA-N2a or LC-MS/MS analyses.

### 5.5. Neuroblastoma Cell-Based Assay

Methanol extracts were screened for the presence of CTXs using the CBA-N2a assay performed following the protocol previously described by Darius et al. [14]. The final concentrations for ouabain/veratridine treatment (OV^+^ conditions) ranged from 85/8.5 to 100/10 µM in order to obtain 90–100% of cell viability against control cells without ouabain/veratridine treatment (OV^−^ conditions). Each sample was tested in two or three independent experiments, with each concentration run in triplicate per plate. Methanol extracts were tested using a serial dilution at 1:2 of eight concentrations ranging from 74 to 9524 or 186 to 23,809 pg dry extract µL^−1^ for SPATT devices deployed in November 2016 or August 2018, respectively.

Absorbance data were fitted to a sigmoidal dose–response curve (variable slope) based on the four-parameter logistic model (4PL), allowing the calculation of raw EC_50_ values expressed in pg dry extract µL^−1^ using the Prism v6.07 software (GraphPad, San Diego, CA, USA). For a more convenient presentation of our results, the CBA-N2a curves and EC_50_ of samples were further converted into ng HP20 resin equiv. µL^−1^. The calibration of the assay was achieved using a P-CTX3C reference material sourced from the Institut Louis Malardé (Papeete, French Polynesia) and that had been quantified gravimetrically. The EC_50_ value obtained for P-CTX3C was 1.84 ± 0.31 fg µL^−1^ (n = 5). The toxin contents (T) of samples were estimated using the formula T = (P-CTX3C EC_50_ / sample EC_50_) and expressed in ng P-CTX3C equiv. g^−1^ HP20 resin. The toxin content per SPATT device was also estimated using the following formula: T × 2.5 or 5 g and expressed in ng P-CTX3C equiv. SPATT^−1^. A Mann–Whitney test was applied to the CBA-N2a data using the Prism v6.07 software (GraphPad, San Diego, CA, USA) in order to compare all the toxin contents obtained in November 2016 vs. August 2018.

The maximum concentration of dry extract (MCE) that does not induce non-specific cytotoxic effects on N2a cells was assessed using a methanol extract obtained from 10 g of non-exposed activated HP20 resin. MCE was found to be higher than 1 × 10^4^ pg dry extract µL^−1^, corresponding to 1 × 10^5^ ng HP20 resin equiv. µL^−1^ (Figure 1). The limit of quantification (LOQ) was thus estimated to be at 0.02 ng P-CTX3C equiv. g^−1^ HP20 resin.

### 5.6. Liquid Chromatography Coupled with Tandem Mass Spectrometry Analyses

LC-MS/MS analyses were conducted on methanol extracts for the detection of several marine biotoxin groups.

#### 5.6.1. Detection Method for P-CTXs

P-CTX analysis [44] was carried out using an LC system (UFLC Nexera, Shimadzu, Kyoto, Japan) coupled to a hybrid triple quadrupole/ion-trap mass spectrometer (API4000Qtrap, Sciex, Redwood City, CA, USA) equipped with a turbo spray^®^ interface. A C_18_ Zorbax Eclipse Plus column (1.8 µm, 50 × 2.1 mm, Agilent Technologies, Santa Clara, CA, USA) was employed at 40 °C and eluted at 400 µL min^−1^ with a linear gradient. Eluent A was water and Eluent B was methanol, with both eluents containing 2 mM ammonium formate and 50 mM formic acid. The elution gradient ran from 78% to 88% B over 10 min and was held for 4 min before re-equilibration over 5 min.

Mass spectrometric detection was operated in positive mode and using scheduled Multiple Reaction Monitoring (MRM) with a detection window of 90 s, with *m/z* values of the transitions listed in Table 4. MRM experiments were established using the following electrospray ionization (ESI) parameters: curtain gas (CG) set at 25, ion spray (IS) at 5500 V, a turbogas temperature (T) of 300 °C, gas 1 (GS1) set at 40, and gas 2 (GS2) set at 60 psi, with an entrance potential (EP) of 10 V.

Data processing and analysis were achieved with the Analyst software (Sciex, Redwood City, CA, USA). Quantification was performed from a linear calibration curve generated from the P-CTX3C standard (Wako chemicals GmbH, Neuss, Germany) using the MRM transition [M + H]^+^/[M + H − H_2_O]^+^. The LOD and LOQ for P-CTX3C were 2 and 6 ng mL^−1^, respectively. For the comparison of retention times, an extract of TB92, a highly toxic *G. polynesiensis* strain provided by the Institut Louis Malardé (Papeete, French Polynesia), was injected in the sequence.

#### 5.6.2. Detection Method for OA and DTXs

OA and DTXs are part of our analytical method for lipophilic toxins, performed in negative ionization mode. This analytical method grouped OA, DTXs, and yessotoxins (YTXs) and was carried out on the same instrument system as described above.

Chromatographic separation was carried out on a C_18_ Kinetex (50 × 2.1 mm, 2.6 µm, Phenomenex, Le Pecq, France), using a mobile phase composed of (A) water and (B) 95% acetonitrile, both containing 2 mM ammonium formate and 50 mM formic acid at a flow rate of 400 µL min^−1^ and maintained at 40 °C. The elution gradient ran from 10% to 50% B over 2 min, to 95% B over the next 4 min and was held for 2 min before re-equilibration.

MRM experiments were carried out in negative mode and the *m/z* selected are listed in Table 4. The ESI parameters were set as follows: CG, 20; IS, −4500 V; T, 550 °C; GS1, 40 psi; GS2, 50 psi; and EP, −13 V. Quantification was performed from linear calibration curves generated from certified standards of OA, DTX2, DTX1, and YTX (National Research Council, Ottawa, ON, Canada). The LOD and LOQ for the standards available were determined with the ordinary least-squares regression data method [45,46] (Table 4).

#### 5.6.3. Detection Methods for Other Toxins

In addition, quantitative targeted analyses (MRM mode) were conducted following the methods previously described for some of the other major marine biotoxin groups: (i) lipophilic toxins, i.e., pectenotoxins (PTXs) and azaspiracids (AZAs) [47]; (ii) cyclic imines, i.e., gymnodimins (GYMs), spirolids (SPXs), and pinnatoxins (PnTXs) [47]; (iii) palytoxin-like toxins, i.e., palytoxin (PLTX), 42-hydroxy-palytoxin (42-OH-PLTX), and ovatoxins (OvTXa to OvTXh) [48]; (iv) maitotoxins (MTX1 to MTX4) [49]; and (v) cyanotoxins, i.e., microcystins (dm-MC-RR, MC-RR, MC-YR, MC-LR, dm-MC-LR, MC-LA, MC-LY, MC-LW, and MC-LF) and nodularin (NOD) [50].

## Figures and Tables

**Table 1:** Dinoflagellate genera observed by light microscopy on macroalgal substrates and semi-quantitative polymerase chain reaction (qPCR) estimates of *Gambierdiscus* species composition identified from window screen (WS) samples, from Anaho Bay in November 2016 (n = 6 for each substrate) and August 2018 (n = 5 for each substrate).

Field Experiment	Dinoflagellate Genera Observed on Macroalgal Substrates	qPCR Analyses of Artificial WS Substrates	
		Cell Density of *Gambierdiscus* spp. ^2^	*Gambierdiscus* Species Identification
**November 2016 ^1^**	*Gambierdiscus* *Ostreopsis* *Prorocentrum*	~2900	*G. polynesiensis* (82%) *G. carpenteri* (17%) *G. caribaeus* (<1%) *G. pacificus* (<1%) *G. toxicus* (<1%)
**August 2018**	*Ostreopsis* *Gambierdiscus*	ND	ND

^1^ Data from Darius, et al. [14]; ^2^ Density expressed in cells 150 cm^−2^. ND = not detected.

**Table 2:** Mean half-maximal effective concentration (EC_50_) values (CBA-N2a) and toxin content estimates for SPATT devices deployed in Anaho Bay in November 2016 and August 2018.

Field Experiment	Deployment Time (days)	Resin Load (g)	Surface of Exposure (cm^2^)	EC_50_^1^ (ng HP20 Resin Equiv. µL^−1^)	Toxin Content (ng P-CTX3C Equiv. g^−1^ HP20 Resin)
November 2016	1	2.5	44	410 ± 78	4.6 ± 0.9
		5		486 ± 52	3.8 ± 0.4
	3	2.5		270 ± 51	7.0 ± 1.2
		5		381 ± 44	4.9 ± 0.6
August 2018	1	2.5	44	14,611 ± 4817	0.14 ± 0.04
		5		4937 ± 966	0.38 ± 0.08
	6	2.5	44	4589 ± 149	0.40 ± 0.01
			71	3661 ± 1391	0.54 ± 0.21
		5	44	2290 ± 410	0.82 ± 0.14
			71	1095 ± 95	1.7 ± 0.2

^1^ Data represent the mean ± SE of values obtained from two or three different SPATT devices, each tested in two to three independent CBA-N2a experiments.

**Table 3:** Estimated OA and DTX1 contents in SPATT devices deployed in Anaho Bay in November 2016 and August 2018, based on LC-MS/MS data.

Field Experiment	Deployment Time (Days)	Resin Load (g)	Surface of Exposure (cm^2^)	Toxin Content ^1^ (ng Toxin SPATT^−1^)	
				OA	DTX1
**November 2016**	1	2.5	44	9.0 ± 1.6	ND ^2^
		5		21.3 ± 4.3	ND
	3	2.5		13.0 ± 1.3	ND
		5		37.0 ± 13.0	4.2 ± 1.6
**August 2018**	1	2.5	44	3.3 ± 0.1	ND
		5		6.2 ± 0.8	ND
	6	2.5	44	18.6 ± 0.2	2.1 ± 0.5
			71	25.4 ± 1.3	2.0 ± 0.1
		5	44	25.4 ± 6.4	3.1 ± 0.8
			71 ^3^	47.0	7.0

^1^ Data represent the mean ± SE of values obtained from two or three different SPATT devices and are expressed in ng of toxin per SPATT device; ^2^ ND: not detected; ^3^ Only one SPATT device was analyzed.

**Table 4:** List of selected *m/z* for the MRM experiments.

Compound	Pseudo Molecular Ion	MRM Transitions (*m/z*)	DP (V)	CE (eV)	LOD (ng mL^−1^)	LOQ (ng mL^−1^)
P-CTX1B	[M + NH_4_]^+^	1128.6/1093.6	105	20		
		1128.6/1075.6 ^1^	105	30		
		1128.6/95.1	105	90		
P-CTX3C & P-CTX3B	[M + NH_4_]^+^	1040.5/1005.6	105	30		
	[M + H]^+^	1023.6/1005.6 ^1^	105	20	2	6
		1023.6/125.1	105	50		
P-CTX4A & P-CTX4B	[M + NH_4_]^+^	1078.6/1043.6	105	30		
	[M + H]^+^	1061.6/1043.6 ^1^	105	20		
		1061.6/125.1	105	50		
2,3-diOH-P-CTX3C	[M + NH_4_]^+^	1074.6/1039.6	105	30		
	[M + H]^+^	1057.6/1039.6 ^1^	105	20		
		1057.6/125.1	105	50		
51-OH-P-CTX3C	[M + NH_4_]^+^	1056.6/1021.6 ^1^	105	30		
	[M + H]^+^	1039.6/1021.6	105	20		
		1039.6/1003.6	105	20		
M-*seco*-P-CTX3C	[M + H]^+^	1041.6/1023.6 ^1^	105	30		
		1041.6/1005.6	105	20		
		1041.6/125.1	105	50		
P-CTX2 & P-CTX3	[M + NH_4_]^+^	1112.6/1077.6	105	20		
		1112.6/1059.6 ^1^	105	30		
		112.6/95.1	105	90		
2-OH-P-CTX3C & 3-OH-P-CTX3C	[M + NH_4_]^+^	1058.6/1023.6 ^1^	105	30		
		1058.6/1005.6	105	20		
		1058.6/125.1	105	50		
OA	[M − H]^−^	803.4/255.1 ^1^	−170	−62	1	3
		803.4/113.1	−170	−92		
DTX2	[M − H]^−^	803.4/255.1 ^1^	−170	−62	1	3
		803.4/113.1	−170	−92		
DTX1	[M − H]^−^	817.5/255.1 ^1^	−170	−68	1	3
		817.5/113.1	−170	−92		
YTX	[M − H]^−^	1141.4/1061.6 ^1^	−120	−48	1	3
		1141.4/855.6	−120	−98		
Homo-YTX	[M − H]^−^	1155.5/1075.6 ^1^	−120	−48		
		1155.5/869.4	−120	−98		
45-OH YTX	[M − H]^−^	1157.5/1077.5 ^1^	−120	−48		
		1157.5/855.5	−120	−98		
45-OH homo YTX	[M − H]^−^	1171.5/1091.5 ^1^	−120	−48		
		1171.5/869.4	−120	−98		
COOH YTX	[M − H]^−^	1173.5/1093.5 ^1^	−120	−48		
		1173.5/855.5	−120	−98		
Homo COOH YTX	[M − H]^−^	1187.5/1107.5 ^1^	−120	−48		
		1187.5/869.4	−120	−98		

^1^ MRM transition used for quantification in each method.

## Supplementary Materials

The following are available online at https://www.mdpi.com/2072-6651/12/5/321/s1, Table S1: Read number and classification levels at 98% identity for the large subunit ribosomal RNA gene region from each window screen sample collected from Anaho Bay (Nuku Hiva Island, French Polynesia) in August 2018. Figure S1: Phylogenetic analysis of large subunit ribosomal DNA (D1-D2 region) sequences obtained from the high-throughput sequencing metabarcoding using Bayesian analyses. Sequences in grey represent the consensus sequence from all reads of each taxonomic assignment. Values at nodes represent Bayesian posterior probability support. Scale bar is substitutions per site.
