# Supplementary material for: Assessment of Ciguatera and Other Phycotoxin-Related Risks in Anaho Bay (Nuku Hiva Island, French Polynesia): Molecular, Toxicological, and Chemical Analyses of Passive Samplers
Source: Toxins (Basel). 2020 May 13;12(5):321. doi: 10.3390/toxins12050321 (PMC7291316; doi:10.3390/toxins12050321)
Supplement: Supplementary file 1 [file toxins-12-00321-s001.pdf]

Assessment of Ciguatera and Other Phycotoxin-Related Risks in Anaho Bay (Nuku Hiva Island, French Polynesia): Molecular, Toxicological, and Chemical Analyses of Passive Samplers

Mélanie Roué, Kirsty F. Smith, Manoella Sibat, Jérôme Viallon, Kévin Henry, André Ung, Laura Biessy, Philipp Hess, Hélène Taiana Darius and Mireille Chinain

Table S1. Read numbers and classification levels at 98% identity for the large subunit ribosomal RNA gene region from each window screen (WS) sample collected from Anaho Bay (Nuku Hiva Island, French Polynesia) in August 2018.

Taxon	WS 1	WS 2	WS 3	WS 4	WS 5	Total	% of Dinophyceae reads
**Total number of reads**	147,971	154,679	157,887	165,974	126,159	752,670	/
**Number of Dinophyceae reads**	27,069	82,778	97,610	62,489	53,923	323,869	100
Amoebophrya sp.	136	2061	2995	2097	325	7614	2.35
Amphidinium cf. *carterae*	16	0	59	47	58	180	1.63
Amphidinium cf. *massartii*	184	349	346	312	330	1521	
Amphidinium fijiensis	0	13	0	0	0	13	
Amphidinium cf. *thermaeum*	444	982	1275	357	516	3574	
Azadinium sp.	0	0	0	20	0	20	0.01
Azadinium polongum	0	0	0	16	0	16	
Biecheleria sp.	3	28	0	19	3	53	0.02
Biecheleriopsis sp.	5	0	0	0	4	9	>0.01
cf. *Bysmatrum* sp.	9	0	13	2	23	47	0.01
Calciodinellum operosum	4	1	18	8	1	32	0.01
Cochlodinium sp.	2706	4295	1186	4280	107	12,574	3.88
Coolia sp.1	31	18	64	41	229	383	0.30
Coolia sp.2	48	26	95	61	344	574	
Dinophysis sp.	12	7	12	59	10	100	0.03
Gambierdiscus carpenteri	338	44	225	87	557	1251	1.78
Gambierdiscus pacificus	505	262	562	0	482	1811	
Gambierdiscus polynesiensis	201	321	1802	307	6	2637	
Gambierdiscus toxicus	20	22	15	0	9	66	
Gymnodinium cf. *dorsalisulcum*	19	131	73	162	11	314	0.12
Gymnodinium sp.	0	53	0	29	0	82	
Gyrodiniellum sp.	32	107	138	328	8	613	0.19
Gyrodinium jinhaense	327	1649	1193	1705	137	5011	1.55
Heterocapsa sp.1	0	3	0	0	0	3	0.08
Heterocapsa sp.2	20	44	79	92	11	246	
Karenia umbella	2	8	17	7	1	35	0.02
Karenia cf. *papilionacea*	0	16	0	0	0	16	
Karlodinium cf. *ballantinum*	0	325	0	109	0	434	0.2
Karlodinium veneficum	2	11	23	10	1	47	
Karlodinium sp.	4	82	0	51	0	137	
Katodinium glaucum	0	16	0	0	0	16	>0.01
Lepidodinium chlorophorum	30	45	115	74	57	321	0.10
Levanderina fissa	6	87	19	67	9	188	0.06
Ostreopsis cf. *ovata*	4233	17,746	16,353	7820	6919	53,071	47.51
Ostreopsis cf. *lenticularis*	463	281	459	409	375	1987	
Ostreopsis cf. *rhodesiae*	7845	34,106	28,841	13,315	14,696	98,803	

Pelagodinium beii	5	53	15	13	15	101	0.03
Pentapharsodinium sp.	0	0	0	0	8	8	>0.01
Peridiniella sp.	14	66	37	200	10	327	0.10
Polykrikos sp.	69	160	225	750	15	1238	0.38
Prorocentrum sp.1	0	36	6	27	0	69	0.35
Prorocentrum sp.2	8	4	15	11	9	47	
Prorocentrum rhathymum	84	129	383	279	123	998	
Prorocentrum triestinum	3	0	14	14	2	33	
Scrippsiella lachrymosa	1	42	84	49	5	181	0.06
Stoeckeria algicida	2	1	7	3	0	13	>0.01
Symbiodinium sp.D	7	112	0	56	19	194	0.20
Symbiodinium sp.B	2	3	383	6	16	410	
Symbiodinium sp.C	5	39	3	0	0	47	
Takayama sp.1	1	8	18	7	0	34	0.02
Takayama sp.2	2	0	35	0	0	37	
Unclassified Dinophyceae reads	9212	19,039	40,408	29,212	28,462	126,333	39.01
Unclassified reads (other Eukaryotes)	120,902	71,901	60,277	103,485	72,236	428,801	/

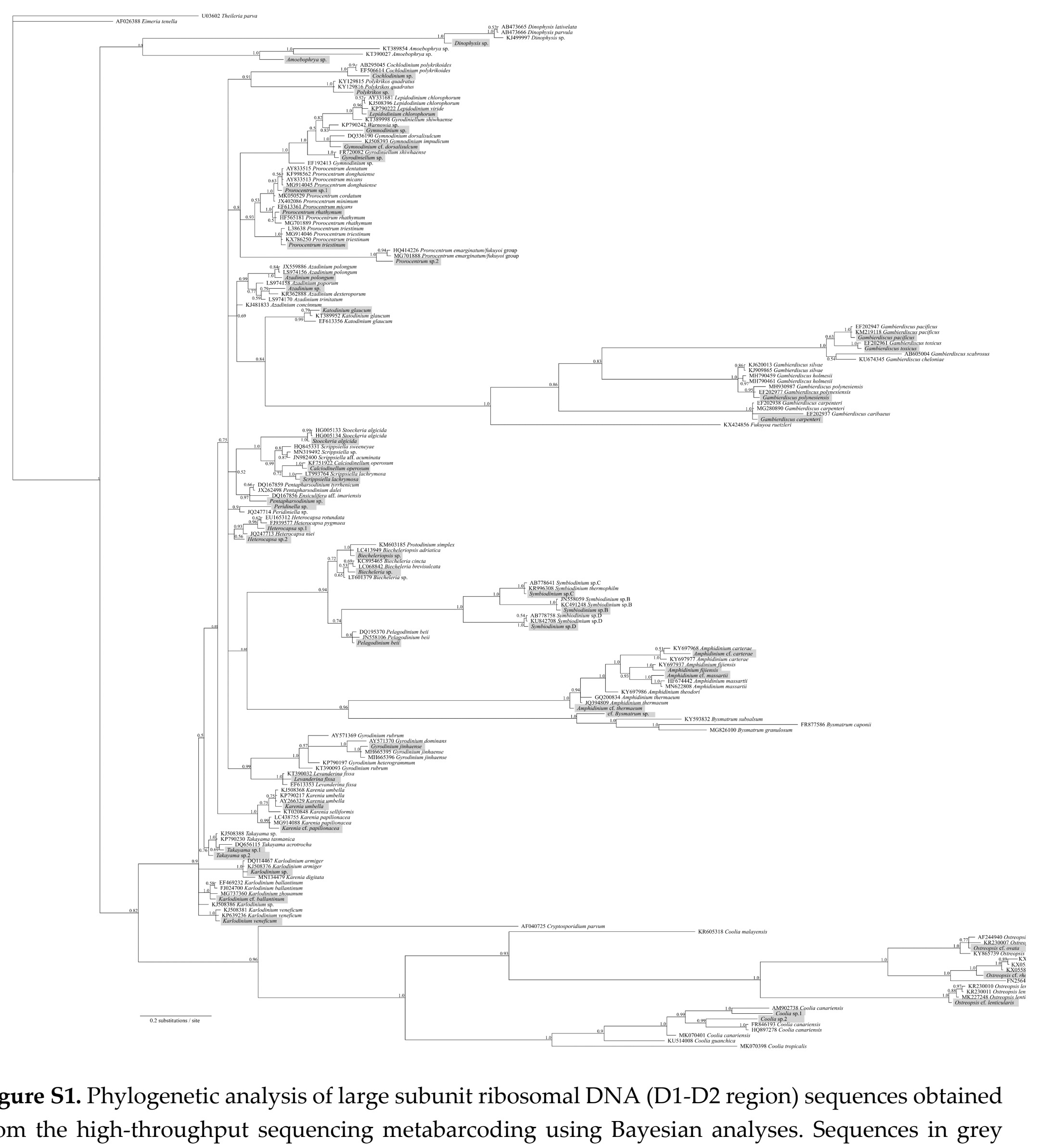

Figure S1. Phylogenetic analysis of large subunit ribosomal DNA (D1-D2 region) sequences obtained from the high-throughput sequencing metabarcoding using Bayesian analyses. Sequences in grey represent the consensus sequence from all reads of each taxonomic assignment. Values at nodes represent Bayesian posterior probability support. Scale bar is substitutions per site.
